# Supplementary material for: Development of Systemic Interventions to Decrease Breast Cancer Risk: A Group Concept Mapping Study
Source: Int J Environ Res Public Health. 2024 Mar 9;21(3):318. doi: 10.3390/ijerph21030318 (PMC10970315; doi:10.3390/ijerph21030318)
Supplement: Supplementary file 1 [file ijerph-21-00318-s001.zip › ijerph-2865129-supplementary.pdf]

TABLE S1 Interventions, sorted by cluster, all in response to the prompt: "One way to reduce breast cancer risk, particularly in disproportionately impacted communities is..." For number of regions across California for which average ratings for interventions appear in the four different quadrants of the Go-Zones: HiI = High Importance, HiF = High Feasibility, LoI = Low Importance, LoF = Low Feasibility.

Cluster	Inter-vention#	Intervention	# Regions in HiI & HiF zones	# Regions in HiI & LoF zones	Total # regions in Hi I zones	# Regions in LoI & LoF zones	# Regions in LoI & HiF zones
**1: Promote Community Self-Determination**							
	1	With community input and leadership, develop strategies to reduce points of sale in communities with high concentrations of retail alcohol outlets.	0	3	3	5	1
	32	Require community input on land use decisions for locations with potential radioactive contamination.	1	4	5	2	2
	52	Create training opportunities for community members to collect high-quality data that monitors air, soil, and water quality that can be used to enforce pollution prevention measures.	2	2	4	3	2
	31	Require full transparency to residents, developers, and other interested parties of potential radioactive contamination in their communities.	4	3	7	1	1
		Cluster 1 averages	**1.8**	**3.0**	**4.8**	**2.8**	**1.5**
**2: Reduce Air, Water & Light Pollution**							
	35	Reduce outdoor night-time light as much as possible, without sacrificing neighborhood safety, using strategies such as lighting only necessary areas, minimizing glare, reducing brightness, and using motion sensors.	0	1	1	8	0

36	Adopt city- or county-wide lighting ordinances which set standards that reduce light pollution.	0	1	1	7	1
57	Implement state-of-the-art street-cleaning methods to protect local air quality and downstream water quality.	0	1	1	5	3
58	Strengthen and expand existing idling regulations for commercial heavy-duty diesel vehicles and passenger cars.?	0	2	2	6	1
59	Facilitate, through requirements and incentives, faster transition to cleaner engine technologies in trucking, shipping, port operations, and trains.?	0	2	2	6	1
67	Move commercial trucking routes away from residential areas.	0	0	0	6	3
73	Expand public transportation systems to underserved neighborhoods that link to essential services, schools, and places of employment.	0	2	2	5	2
78	Expand resources and infrastructure for the purchase and use of electric vehicles across the economic spectrum.	0	1	1	8	0
70	Require cities and counties to increase useable sidewalks, safe intersections, adequate lighting, and well-connected walking routes, with leadership from communities.	1	1	2	4	3
54	Adequately fund groundwater testing in areas where people rely on wells for their water source and provide filters when contamination is identified.	2	3	5	0	4
55	Work with agriculture and state/local agencies to adopt aggressive measures to reduce agricultural water pollution from pesticides, fertilizer, and livestock operations.	2	5	7	0	2
	Cluster 2 averages	**0.5**	**1.7**	**2.2**	**5.0**	**1.8**

3: Increase Access to Healthy Food and Exercise

22	Create buffer zones that limit the density of unhealthy fast-food outlets around schools.	0	2	2	7	0
37	Provide free blackout curtains, sleep masks, and information on other strategies to reduce exposure to night-time light to residents living near facilities with bright outside lighting.	0	1	1	7	1
68	With community input and leadership, encourage bike use by expanding the network of bike lanes and access for bikes on public transportation.	0	1	1	7	1
69	Expand affordability and access to bike share networks in communities of color and low-income areas, while ensuring bikes do not hinder access to sidewalks.	0	0	0	7	2
20	Implement local initiatives that support affordable fresh produce at corner stores, bodegas, and community markets.	1	4	5	1	3
71	Create safe routes for children in underserved communities to walk/bike to school through construction of new bicycle lanes and sidewalks, with community input and leadership.	1	0	1	5	3
75	Provide funding for public spaces in underserved neighborhoods (for example, community centers and outdoor spaces).	1	1	2	2	5
26	Develop community kitchens that bring seniors together to cook and eat healthy food that they can also bring home.	2	1	3	4	2
72	Expand public transportation systems to underserved neighborhoods that link to essential services, schools, and places of employment.	2	1	3	5	1
77	Create healthier communities by planting more trees, installing benches, and funding public art projects in existing and new public spaces, with community input and leadership.	3	1	4	0	5
21	Provide public support to share locally grown excess food (for example, food swaps) and ensure farmer's markets are affordable to local communities with limited access to fresh produce.	5	1	6	1	2
19	Provide support for community gardens (for example, education and training, seeds, clean soil, natural pest management, and gardening tools).	7	0	7	0	2
	Cluster 3 averages	**1.8**	**1.1**	**2.9**	**3.8**	**2.3**

**4: Incorporate Healthy Lifestyle Activities in Schools & Communities**						
49	Collaborate with and fund local organizations to provide physical activity opportunities in after school, camp, or recreation programs.	0	1	1	4	4
25	Develop school gardens that provide gardening, nutrition, and cooking curricula at multiple schools, ranging from K-12, across a school district.	2	0	2	2	5
46	Work with school districts to ensure K-12 schools have the resources they need to offer universal physical education classes.	2	1	3	1	5
51	Increase free physical activity options in underserved communities by creating culturally tailored programs across the lifespan (for example, walking programs and Zumba in the Park).	2	0	2	3	4
76	Provide incentives for schools to offer community programming (for example, physical activity and food swaps) after hours and on weekends.	2	0	2	1	6
23	Incorporate student input to provide healthy school lunch programs that students are likely to eat.	3	0	3	2	4
24	Provide free healthy breakfast and lunch programs without income requirements to K-12 students when school is in session and during summer breaks.	4	0	4	0	5
	Cluster 4 averages	**2.1**	**0.3**	**2.4**	**1.9**	**4.7**
**5: Provide Preventive Education & Resources**						
48	Provide continuing education and trainings for K-12 teachers to incorporate body movement that amplifies learning.	1	0	1	1	7
62	Subsidize childcare, starting from infant care, to expand the percentage of working families who receive benefits.	1	2	3	4	2
9	Provide free phone or in-home breastfeeding support to new parents from hospital/health department lactation staff.	2	0	2	1	6

10	Provide financial and educational resources to community groups to make available a resource directory of local lactation support services for new parents.	2	1	3	2	4
50	Develop workplace physical activity programs promoting movement (for example, taking stairs, active commutes, and on-site exercise classes).	2	0	2	1	6
65	Work with community groups to provide culturally tailored education and advocacy on how to reduce the disproportionate impact of breast cancer risk factors for under-resourced communities, including rural, tribal, and communities of color.	6	0	6	0	3
	Cluster 5 averages	**2.3**	**0.5**	**2.8**	**1.5**	**4.7**
**6: Create Interventions Related to Reproductive and Women's Health**						
5	Encourage public health departments to promote breastfeeding through multilingual social media and Public Service Announcement (PSA) campaigns.	0	1	1	2	6
11	Improve quality and access to in-person and virtual one-on-one and group peer support breastfeeding programs.	2	1	3	0	6
83	Create, fund, and promote culturally tailored smoking cessation programs with online, phone, and text accessibility.	2	1	3	5	1
7	Create breastfeeding-friendly workplaces with a special focus on work sectors that face unique challenges (for example, farmworkers).	3	0	3	3	3
43	Provide education to the general public about the natural process of menopause and ways to manage symptoms without hormone therapy.	4	1	5	0	4
8	Train community health educators, doulas, and promatoras to offer culturally tailored and affordable lactation support to address the unique challenges of communities with lower rates of breastfeeding.	5	0	5	1	3

3	Make educational materials on the links between alcohol and breast cancer easily available at health clinics, community centers, and via social media.	6	1	7	0	2
	Cluster 6 averages	**3.1**	**0.7**	**3.9**	**1.6**	**3.6**
**7: Implement Preventive Health Measures**						
34	Educate night shift workers who are exposed to night-time light about the benefits of exposure to daylight and encourage them to get regular exposure to daylight.	1	0	1	2	6
6	Require all healthcare plans, including MediCal, to fully cover lactation support and all breastfeeding equipment for new parents.	2	5	7	0	2
45	Work with professional medical organizations to develop guidance for health care professionals on risks and benefits of hormone replacement therapy use to control the symptoms of menopause.	2	1	3	2	4
64	Require ongoing anti-racism training for medical providers, medical students, and health care systems to reduce implicit bias and ensure fair and equitable medical care.	5	1	6	1	2
42	Create culturally tailored educational programs, in collaboration with agricultural workers, on their workplace rights, safety regulations, and how to advocate for stronger protections to reduce hazardous exposures.	6	1	7	0	2
44	Add breast cancer risk to the existing risk and benefit information for oral contraceptives and other hormone-based birth control methods.	6	2	8	0	1
82	Include the connection between tobacco use and breast cancer risk in the California K-12 public school tobacco-prevention curriculum.	7	0	7	0	2
84	Create culturally tailored anti-smoking and anti-vaping messages with and for youth and communities of color.	7	0	7	0	2

	Cluster 7 averages	4.5	1.3	5.8	0.6	2.6
**8: Reduce Hazardous Workplace Exposures**						
41	Compare salons certified under the CA Healthy Nail Salon Recognition Program to those that are not part of the program on reduced exposure to breast cancer risk factors.	0	0	0	5	4
28	Avoid duplicate imaging procedures by establishing best practices for health care provider access to previous medical images across health care systems and community clinics.	1	3	4	1	4
29	Establish best practices and train all personnel potentially exposed in health care settings (including, for example, cleaning and other support staff) on how to minimize radiation exposure from medical devices.	6	0	6	1	2
66	Require the state cancer registry to collect information on social factors that impact health, including race, household income, and occupation.	6	0	6	1	2
27	Ensure all medical radiological equipment complies with state standards to minimize radiation exposures from medical imaging.	6	0	6	0	3
	Cluster 8 averages	**3.8**	**0.6**	**4.4**	**1.6**	**3**
**9. Require Transparency & Best Practices to Reduce Harmful Exposures**						
33	Identify and implement best practices to mitigate workplace night-time light exposures while maintaining a safe work environment.	1	1	2	5	2
47	Implement policies at the state, county, and school board levels to limit screen time in childcare centers and after-school programs.	1	2	3	6	0
63	Declare racism a public health crisis at the city, county, and state levels to address the root causes of health disparities (for	1	0	1	4	4

	example, cumulative chemical exposures, poverty, and social stressors).					
12	Pass state right-to-know laws requiring ingredient disclosure for consumer products, such as food packaging, electronics, or school and art supplies.	2	5	7	0	2
38	Develop best practices to monitor and reduce electromagnetic field exposures for occupations of high concern (for example, power grid/telecommunication workers).	3	3	6	3	0
18	Require the state to develop and publish a list of endocrine disrupting chemicals to inform consumer choice and policies to reduce exposure.	4	2	6	0	3
30	Implement best practices to minimize cumulative exposure to radiation in non-medical workplaces.	4	1	5	0	4
39	Develop, with worker input, industry-wide best practices and policies to reduce workplace exposures to chemicals and other breast cancer risk factors.	6	1	7	1	2
	Cluster 9 averages	**2.8**	**1.9**	**4.6**	**2.4**	**2.1**
**10. Promote Policies to Reduce Alcohol & Tobacco Use**						
4	Expand the ban on advertising of alcoholic beverages to include beer and wine on all state-funded university and community college campuses in California.	3	1	4	0	5
80	Ban tobacco sales, including vaping products, in all California pharmacies.	3	3	6	0	3
17	Require all public and private colleges in California to adopt 100% tobacco-free and vape-free campus policies.	4	2	6	0	3
2	Prohibit alcohol advertising within 500 feet of schools, playgrounds, recreation centers or facilities, childcare centers, or libraries.	5	0	5	0	4
81	Work with local, county, and tribal governments to ban tobacco-product advertising that targets youth and communities of color.	5	1	6	0	3

79	Deny new permits for tobacco retail sales within 1000 feet of schools, including vaping products.	6	0	6	0	3
	Cluster 10 averages	**4.3**	**1.2**	**5.5**	**0.0**	**3.5**

11. Regulate Hazardous Products

53	Reform and develop CalEPA program(s) to help businesses generate less solid and hazardous waste, use fewer toxic chemicals, conserve water and energy, and reduce pollution.	2	4	6	1	2
56	Prohibit contaminated wastewater from fossil fuel drilling (for example, fracking) from being used to irrigate crops.	2	5	7	0	2
74	Reduce the use of products containing harmful chemicals in low-income housing (for example, pesticides, cleaning products, carpeting, paint, furniture, and insulation).	2	3	5	0	4
13	Implement state and local government purchasing ordinances that reduce or eliminate the governmental purchase of products containing toxic chemicals.	3	3	6	0	3
61	Develop municipal ordinances to ban pesticides in favor of less toxic pest-management in parks, recreation fields, golf courses, schools, and public spaces.	3	4	7	0	2
16	Develop policies to prevent toxic products being sold or disposed of in communities of color or low-income communities, particularly after laws are passed to disclose, reduce, or eliminate chemicals in consumer products.	4	2	6	1	2
40	Analyze effectiveness of the California regulations requiring petroleum refineries to replace toxic chemicals with non-hazardous alternatives and engage workers and the local community in these efforts.	4	2	6	0	3
14	Ban the use of hazardous chemicals in food packaging sold in the state (for example, BPA and phthalates) and ensure replacement with safer alternatives.	5	2	7	0	2

15	Enact policies at the state/local level that ban hazardous chemicals in consumer products (for example, cleaning products, textiles, and electronics) and ensure replacement with safer alternatives.	5	3	8	0	1
60	Eliminate use of cleaning products containing hazardous chemicals in schools, day care centers, and other buildings open to the public to reduce unsafe exposures, especially for children and workers.	7	1	8	0	1
	Cluster 11 averages	3.7	2.9	6.6	0.2	2.2
